# Supplementary figures and images for: Inflammatory bowel disease biomarkers of human gut microbiota selected via different feature selection methods
Source: PeerJ. 2022 Apr 25;10:e13205. doi: 10.7717/peerj.13205 (PMC9048649; doi:10.7717/peerj.13205)

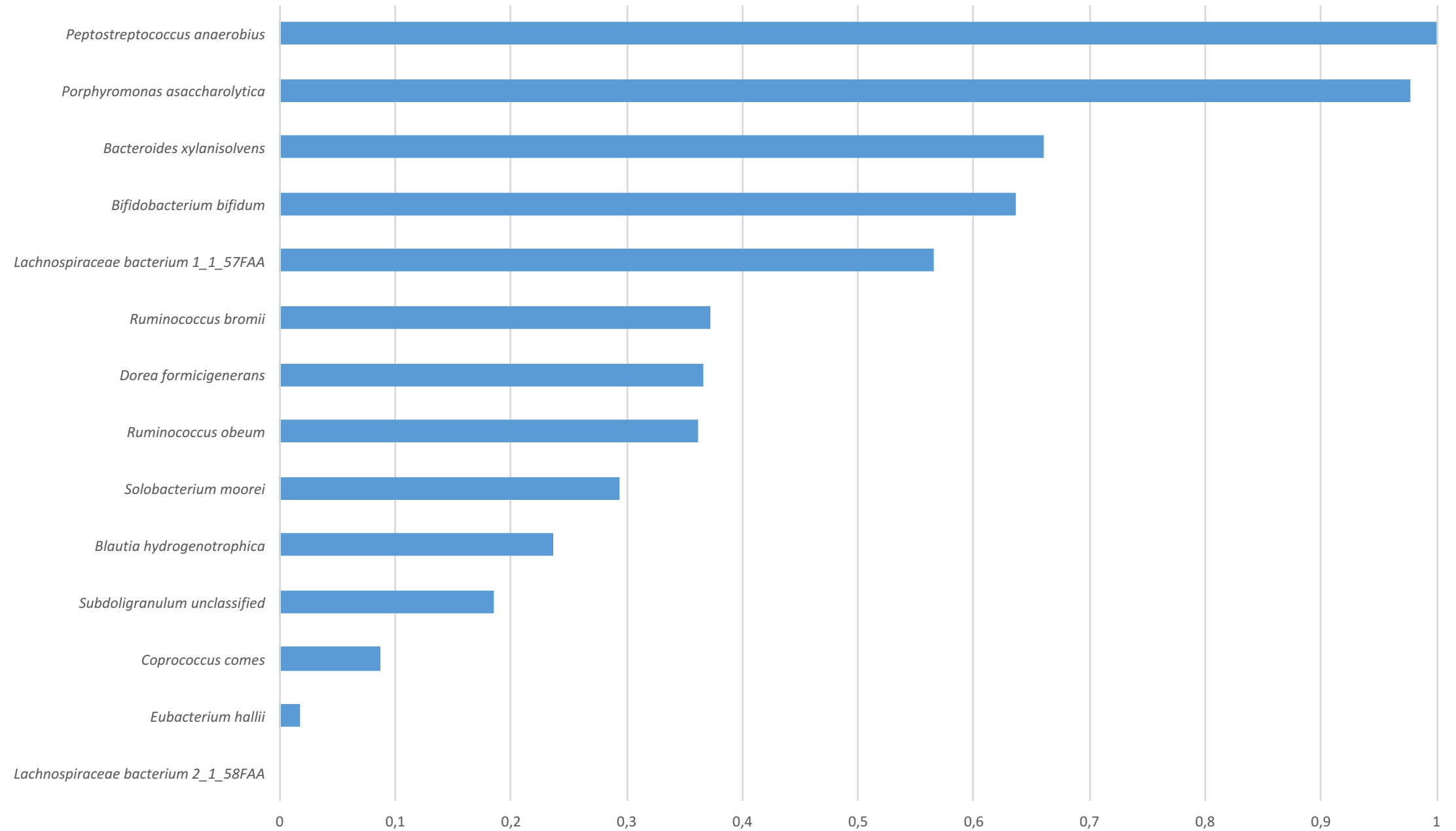

Supplement: Supplemental Information 4 [file peerj-10-13205-s004.pdf]

**A**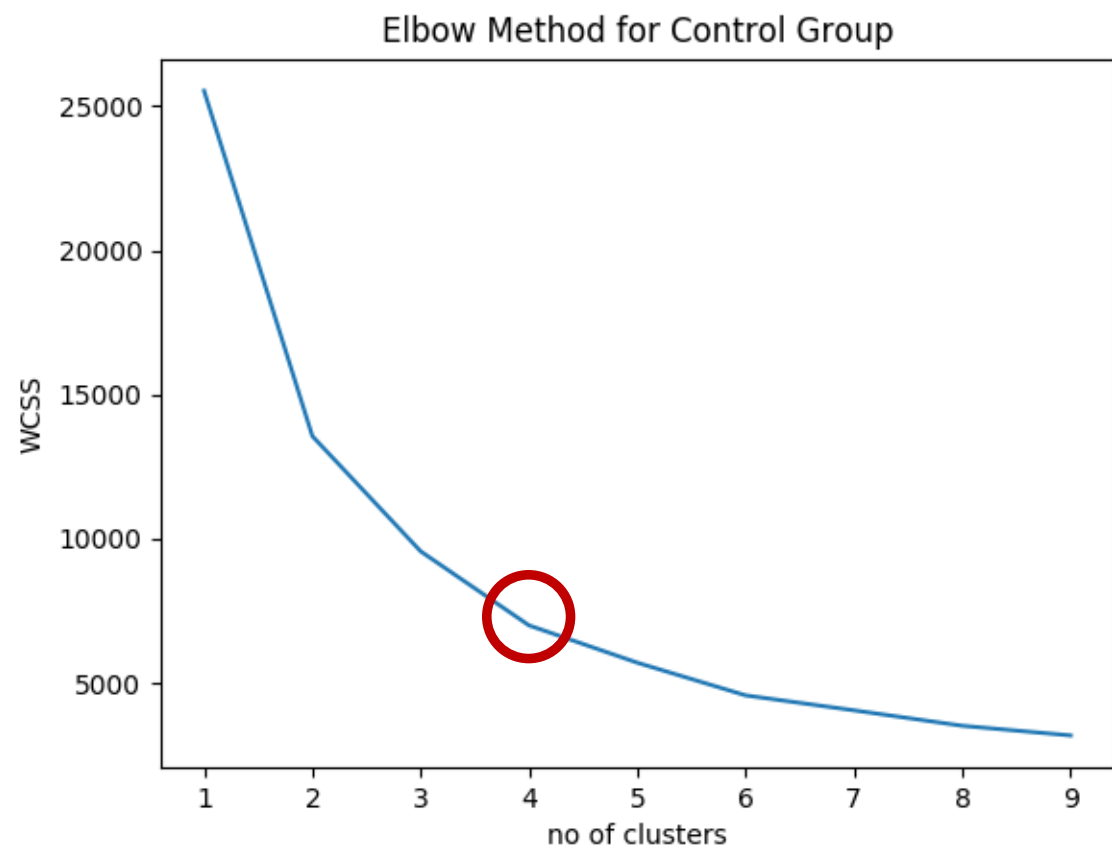**B**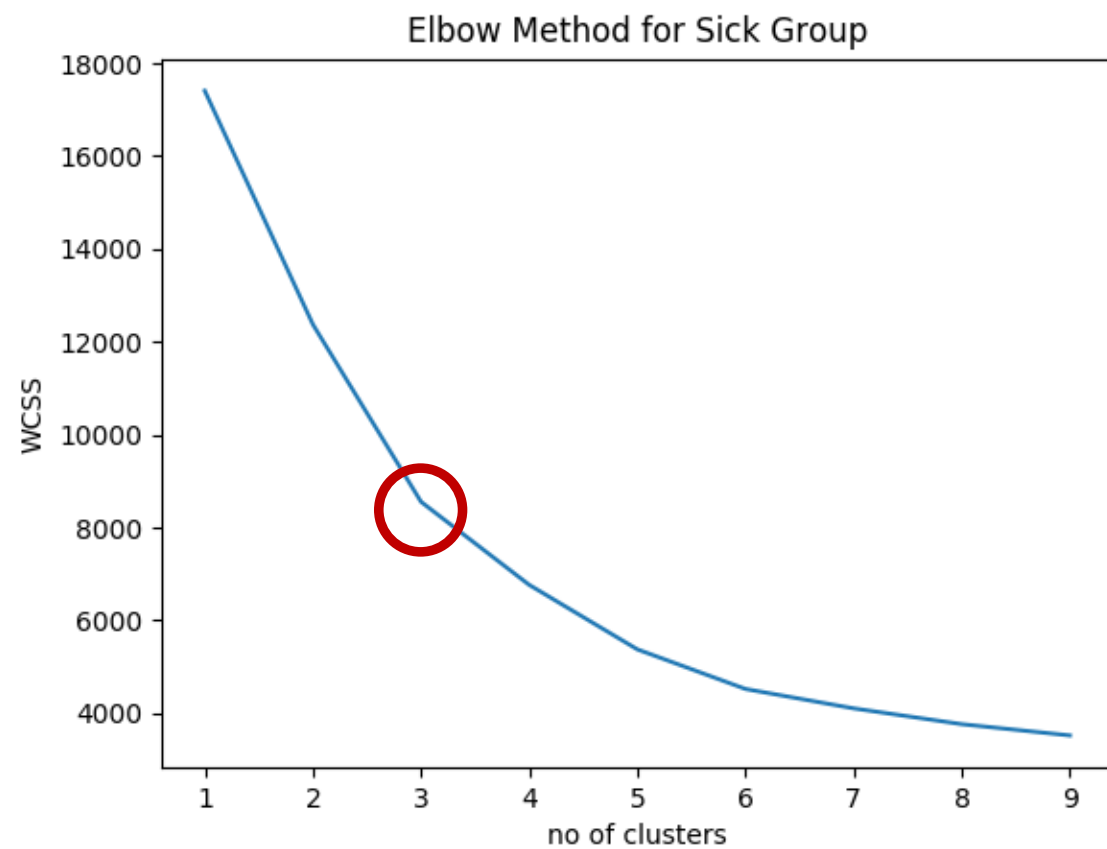

Supplement: Supplemental Information 6 [file peerj-10-13205-s006.pdf]
